# Direct Anterior vs. Posterior Approach in Simultaneous Bilateral Total Hip Arthroplasty: A Meta-Analysis

**DOI:** 10.7759/cureus.75795

**Published:** 2024-12-16

**Authors:** Elcio Machinski, Liron Leibovitch, Jae Yong Park, Iqbal F Sayudo, André Fernandes, Tom Liba, Rodrigo Arruda Conde, Pedro Henrique Cury Tonon, Caio Veloso Gusmão

**Affiliations:** 1 Orthopaedics, State University of Ponta Grossa, Ponta Grossa, BRA; 2 Department of Medicine, Azrieli Faculty of Medicine, Bar-Ilan University, Safed, ISR; 3 Medicine and Surgery, Imperial College London, London, GBR; 4 Department of Medicine, Syiah Kuala University, Banda Aceh, IDN; 5 Trauma and Orthopaedics, Lewisham and Greenwich National Health Services (NHS) Trust, London, GBR; 6 Department of Medicine, Fundación Barceló, Buenos Aires, ARG; 7 Department of Orthopaedics and Traumatology, Technical Educational Foundation Souza Marques, Rio de Janeiro, BRA

**Keywords:** direct anterior approach, hip, hip replacement, posterior approach, simultaneous bilateral hip arthroplasty, total hip arthroplasty approaches

## Abstract

Simultaneous bilateral total hip arthroplasty (SimBTHA) offers benefits such as reduced hospital stay and costs for patients with bilateral hip disease. However, the optimal surgical approach remains uncertain. This study aimed to compare the perioperative outcomes of SimBTHA performed via the direct anterior approach (DAA) versus the posterior approach (PA). A systematic review and meta-analysis were conducted, including studies reporting outcomes of SimBTHA using DAA and PA. The primary outcome was the incidence of allogeneic blood transfusions, while secondary outcomes included blood loss and surgical complications, such as dislocations, periprosthetic fractures, and infections. Six studies were included, analyzing 944 patients, with 372 undergoing SimBTHA via DAA and 572 via PA. No significant difference was observed in the number of allogeneic blood transfusions between the two approaches (RR = 1.04; 95% CI: 0.76 to 1.43; p=0.63). DAA was associated with significantly lower blood loss compared to PA (MD = -31.51 mL; 95% CI: -43.07 to -19.94 mL; p=0.07). However, there was no significant difference in the rates of surgical complications between the two groups (RR = 0.63; 95% CI: 0.32 to 1.26; p=0.12). While DAA showed a benefit in reducing blood loss, it did not demonstrate superiority over PA regarding transfusion rates or surgical complications. These findings highlight the need for further randomized controlled trials with standardized methodologies and longer follow-up periods to better assess the optimal approach for SimBTHA.

## Introduction and background

Total hip arthroplasty (THA) is one of the most prevalent and successful orthopedic approaches of the 20th century [1]. Around 20% of patients seeking this type of procedure suffer from bilateral involvement [2], and up to 85% of patients undergoing unilateral THA will eventually require contralateral THA [3]. Simultaneous bilateral THA (SimBTHA) involves addressing both hip joints during a single surgical procedure with just one anesthetic administration, which can reduce hospital stay and overall surgical costs [4,5].

In addition, several surgical approaches are available for THA [6], and choosing the correct one is essential for optimizing the postoperative period [7]. The direct anterior approach (DAA) stands out for its perioperative advantages, including reducing surgical time, minimizing invasiveness, and promoting faster recovery with less muscle damage and lower postoperative pain [8]. During the DAA, positioning the patient in the supine position offers advantages by allowing simultaneous exposure of both hip joints [9,10]. This simplifies bilateral incisions, eliminating the need to change the surgical field or reposition the patient during the procedure and reducing trauma to the contralateral region, contributing to a more effective postoperative recovery [9].

Although the DAA can enhance postoperative recovery in SimBTHA, this approach often generates concern among surgeons due to the increased risks of blood loss and complications [11]. Therefore, this study aims to conduct a systematic review and meta-analysis to evaluate perioperative outcomes, including allogeneic transfusions, blood loss, and surgical complications of SimBTHA performed via DAA compared to the posterior approach (PA).

## Review

Methods

This systematic review and meta-analysis adhered to the guidelines outlined in the Cochrane Collaboration Handbook for Systematic Review of Interventions [12] and the Preferred Reporting Items for Systematic Reviews and Meta-Analysis (PRISMA) Statement Guidelines [13]. Details of the protocol for this systematic review were registered on PROSPERO (CRD42024585561).

Eligibility Criteria

The eligibility criteria were as follows: (1) peer-reviewed studies, whether prospective or retrospective; (2) reporting on patients who underwent SimBTHA via DAA in both hips compared to PA; (3) reporting any outcome of interest. We excluded studies that (1) were animal or cadaveric studies, case reports, case series, conference abstracts, comments, or editorials; (2) lacked a control group; (3) did not specify the SimBTHA surgical approach. There were no restrictions regarding the date or language of publication.

Search Strategy

We conducted a comprehensive search of electronic databases, including MEDLINE, Embase, and the Cochrane Library, using the following search strategy: ("Hip Replacement" OR "Hip Arthroplasty" OR "Hip Joint Replacement" OR "Hip Joint Arthroplasty" OR THA OR THR) AND (simultaneous OR bilateral OR double OR SimBTHA OR "single-anesthetic" OR "single anesthetic" OR 1-stage OR "one-stage" OR "one stage") AND (anterior OR DAA OR DA).

Data Extraction

After the initial screening, we organized an electronic spreadsheet to collect relevant data systematically. Three authors (I.F.S., P.H.C.T., R.A.C.) extracted the following information from the studies: (1) publication details (first author, publication year, study design); (2) baseline characteristics of the patients, including the number of patients, sex, age, body mass index (BMI), diagnosis, and follow-up duration; (3) primary and secondary outcomes.

For the primary outcome, blood transfusions, we recorded the total number of patients who received allogeneic red blood cell transfusions in each cohort. Blood loss was documented as reported in each study. Based on the criteria established by Chen et al. [14], surgical complications were defined as dislocations, periprosthetic fractures, and infections (either periprosthetic or at the surgical site).

Data Analysis

Treatment effects were measured with 95% confidence intervals. Heterogeneity was evaluated using the I² statistic, with values over 35% considered significant. If this threshold was surpassed, we conducted a sensitivity analysis using the leave-one-out approach. Mean difference (MD) was employed for continuous outcomes, while binary outcomes were analyzed using risk ratios (RR). All statistical analyses were carried out using R Software (version 4.4.0) using the R package 'meta' [15].

Quality Assessment

Two authors (E.M. and L.L.) independently assessed the quality of the included studies using version 2 of the Cochrane Risk Of Bias In Non-randomized Studies - of Interventions (ROBBINS-I) [16]. Disagreements between the two reviewers were resolved through consensus. 

Results

Study Selection

**Figure 1:**
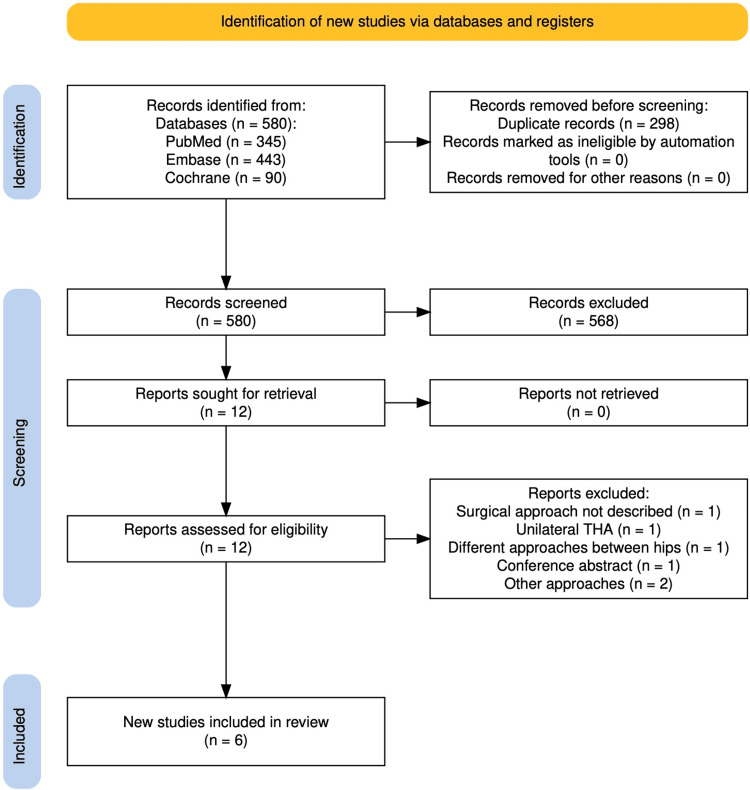
PRISMA flow diagram of included studies PRISMA: Preferred Reporting Items for Systematic Reviews and Meta-Analyses

The initial search identified 878 articles. After eliminating 298 duplicates, 2,017 unique articles remained for screening. Of these, 580 were excluded following a review of titles and abstracts. A full-text assessment of 12 studies, based on the inclusion and exclusion criteria, resulted in the inclusion of 6 studies [9,14,17-20] (Figure 1).

Patients Baseline Characteristics

A total of 944 patients were analyzed, with 372 undergoing SimBTHA via DAA and the remaining 572 receiving SimBTHA via PA. The BMI values were consistent across studies, with a mean of 25.08 kg/m² for the DAA group and 25.57 kg/m² for the PA group. The mean age was 55.04 years for the DAA group and 55.74 years for the PA group. All studies were retrospective cohorts. Additional details are available in Table 1.

**Table 1:** Baseline characteristics of the included studies † Mean ± standard deviation or mean (range). DAA: Direct Anterior Approach; DDH: Developmental Dysplasia of the Hip; F.U.: Follow-up; IJD: Inflammatory Joint Disease; NA: Not Available; OA: Osteoarthritis; ONFH: Osteonecrosis of the Femoral Head; PA: Posterior Approach.

Study	Design	Patients, n (DAA)	Patients, n (PA)	Women, %(DAA)	Women, %(PA)	Age^†^, years (DAA)	Age^†^, years (PA)	BMI^†^, kg/m^2^ (DAA)	BMI^†^, kg/m^2 ^(PA)	Diagnosis	F.U, months	Key conclusions
Chen 2020 [9]	Retrospective	46	43	67.4	67.4	59.6 ± 6	60.2 ± 5	22.72 ± 3	21.67 ± 2.9	OA, ONFH, DDH	DAA: 25.3 PA: 27.6	DAA reduces pain and speeds recovery
Chen 2023 [14]	Retrospective	73	162	90.4	91.3	42.8 ± 8.6	43.3 ± 9.7	24.4 ± 3.4	24.4 ± 3.4	NA	6	DAA shortens surgery and hospital stay
Sarpong 2022 [17]	Retrospective	53	138	NA	NA	NA	NA	NA	NA	OA, ONFH, IJD	NA	No significant transfusion rate differences between DAA and PA
Watts 2016 [18]	Retrospective	19	21	26.6	42.9	53.8 (31-70)	54.2 (40-69)	25.4 (17-35)	27.6 (20-38)	OA, ONFH, DDH, IJD, PTA	3	DAA and PA show similar 90-day result
Micicoi 2019 [19]	Retrospective	55	82	49	55	63 (24-80)	65 (20-80)	24.9 (16-34)	26.3 (15-38)	OA, ONFH, IJD	3	DAA lowers operative time and stay
Torres-Ramirez 2024 [20]	Retrospective	126	126	54	54	56 (20-77)	56 (24-75)	28 (18-49)	27.9 (17-42)	NA	36.7	DAA shortens surgery and discharge time

Quality Assessment

**Figure 2:**
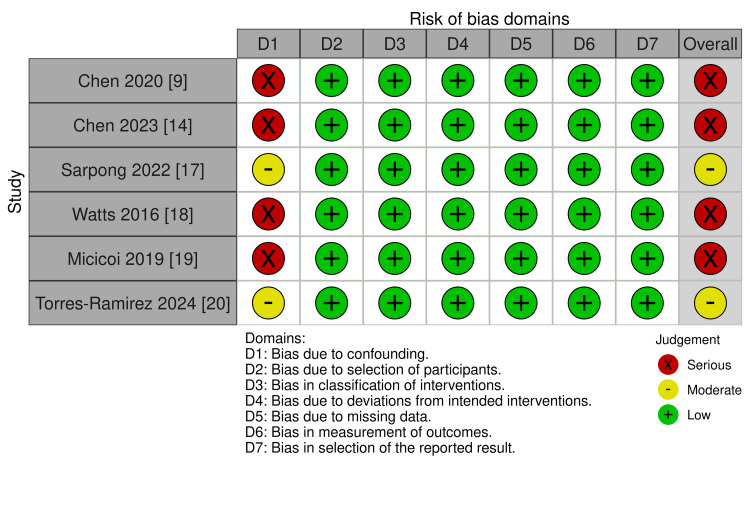
Quality assessment of the included studies (ROBBINS-I) D1: bias due to confounding; D2: bias due to selection of participants; D3: bias in classification of interventions; D4: bias due to deviation from intended interventions; D5: bias due to missing data; D6: bias in the measurement of outcomes; D7: bias in the selection of the reported result ROBINS-I: Risk Of Bias In Non-randomized Studies – of Interventions

The ROBINS-I assessment identified an overall serious risk of bias due to confounding in four of the included studies, while Sarpong et al. [17] and Torres-Ramirez et al. [20] were rated as having a moderate risk of bias. Additional details are provided in Figure 2.

Allogeneic Blood Transfusions

**Figure 3:**
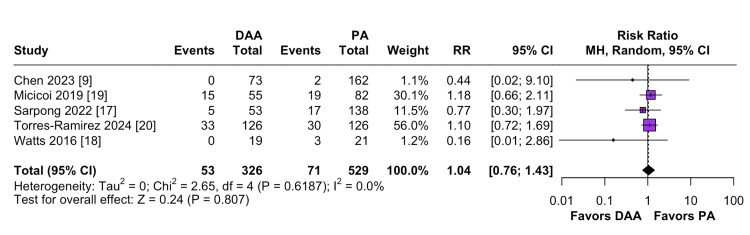
Comparison of allogeneic blood transfusions DAA: Direct Anterior Approach; PA: Posterior Approach

**Figure 4:**
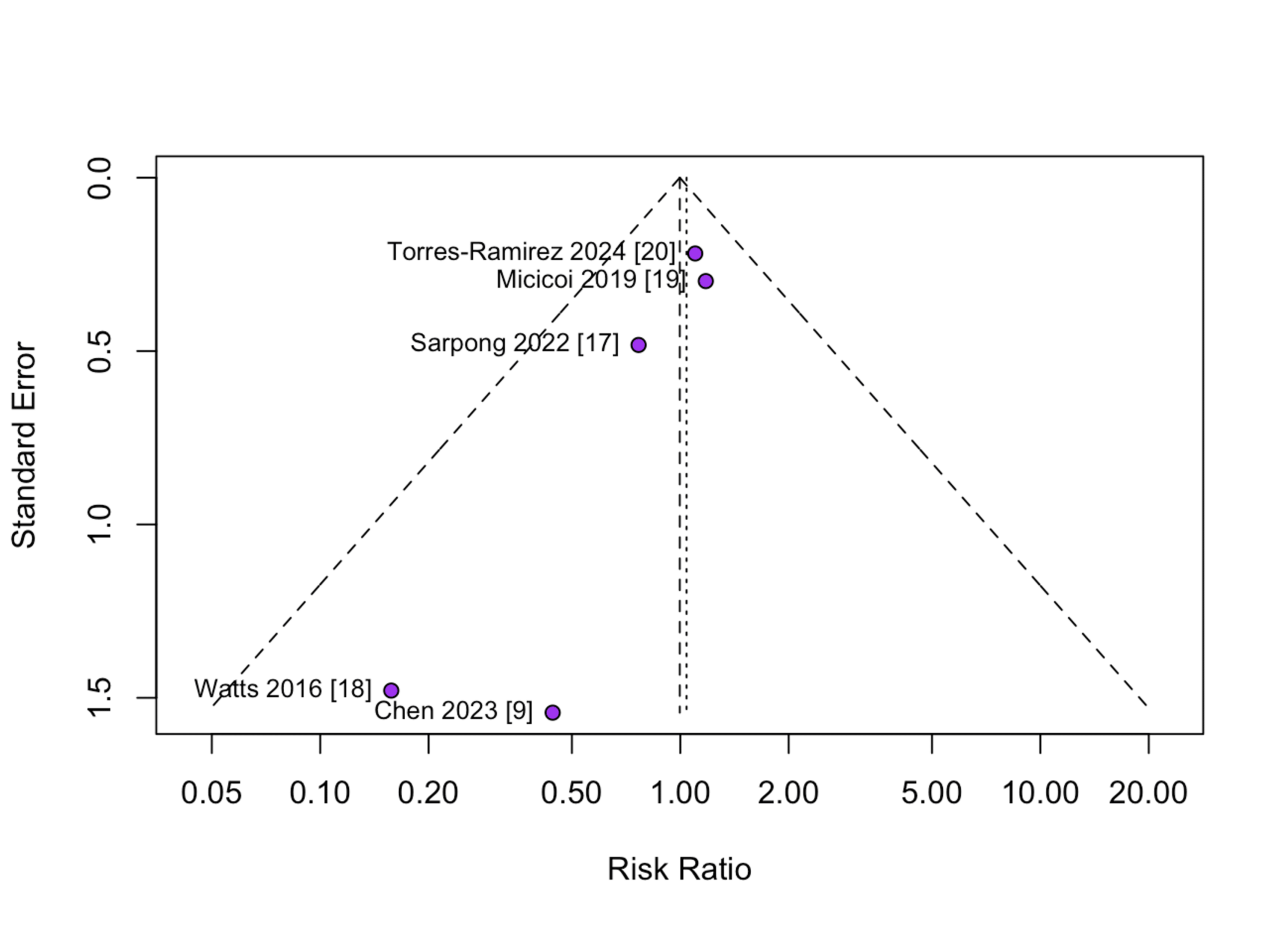
Funnel plot for the analysis of allogeneic blood transfusions, displaying effect sizes (risk ratios) plotted against precision (represented by 1/standard error)

In a cohort of 855 patients, a random effects model analysis found no significant difference between DAA and PA in the total number of patients receiving allogeneic blood transfusions (RR = 1.04; 95% CI: 0.76 to 1.43; P=0.61; I² = 0%; Figure 3). Funnel plot analysis (Figure 4) showed mild asymmetry.

Blood Loss

**Figure 5:**
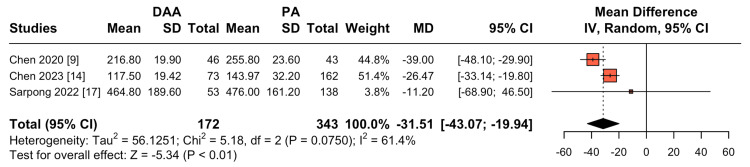
Comparison of blood loss DAA: Direct Anterior Approach; PA: Posterior Approach

Three studies reported data on blood loss. The overall analysis reveals a statistically significant reduction in blood loss with DAA (MD = -31.51; 95% CI: -43.07 to -19.94; P=0.07; I² = 61%; Figure 5).

Surgical Complications

**Figure 6:**
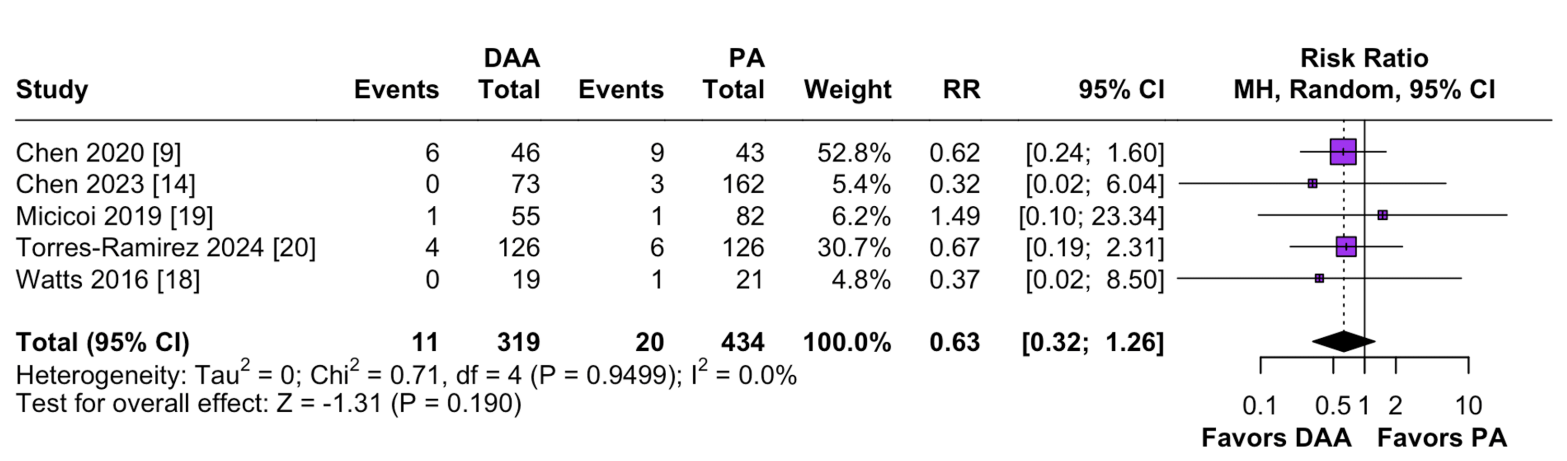
Comparison of surgical complications DAA: Direct Anterior Approach; PA: Posterior Approach

Four studies were included in the surgical complications analysis, which revealed no significant difference between the two approaches (RR = 0.63; 95% CI: 0.32 to 1.26; P=0.94; I² = 0%; Figure 6).

Sensitivity Analysis

**Figure 7:**
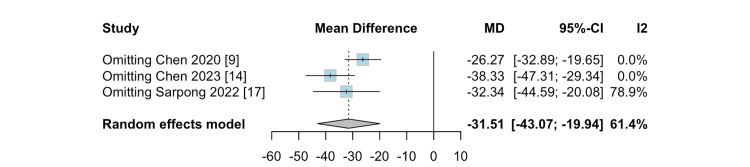
Leave-one-out analysis for blood loss

A leave-one-out analysis on blood loss revealed that omitting Chen et al. [9] or Chen et al. [14] reduced the heterogeneity to zero, indicating that these studies contribute significantly to the overall variability in the analysis. These results are shown in Figure 7.

Discussion

We conducted a systematic review and meta-analysis comparing two surgical approaches, SimBTHA-DAA and PA, focusing on the need for allogeneic transfusion, blood loss, and surgical complications. Our main findings were: (1) no differences in allogeneic transfusion between the two approaches; (2) DAA was superior to PA in reducing blood loss; and (3) no significant differences in surgical complications between the approaches.

The incidence of transfusions in THA is 17% [21], but the risk is higher in SimBTHA [22], where large blood transfusions increase the likelihood of infections, venous thromboembolism, and even mortality [22,23]. Our study found no significant difference between DAA and PA regarding the necessity of blood transfusions. However, Chen et al. reported a significantly lower total transfusion volume in the DAA group compared to PA [9]. Similarly, Parvizi et al. and Jin et al. observed comparable findings when comparing DAA to the direct lateral approach (DLA) and posterolateral approach (PLA), respectively [24,25]. Other factors, such as blood management strategies like the use of tranexamic acid and patient-specific variables like preoperative hemoglobin levels, play a more significant role in the risk of transfusions than the surgical approach alone [17].

We observed less blood loss in SimBTHA via DAA compared to PA, which may be attributed to the muscle-sparing nature of DAA, with reduced soft tissue injury [25]. Operative time is another key factor, as longer durations are associated with higher rates of adverse events, increased blood loss, and a greater need for transfusions [26,27]. Two of the studies [9,14] included in our analysis reported shorter operative times, which may have contributed to reduced blood loss in these cases. However, a network meta-analysis revealed that DAA could be associated with more blood loss than PLA and DLA, possibly due to its steep learning curve [28]. This curve can prolong operative time and increase bleeding, as inadequate exposure may lead to muscle damage, particularly to the tensor fascia latae [28,29].

In our study, we did not find a significant reduction in complications when comparing different surgical approaches. However, it's important to note that we did not account for lateral femoral cutaneous nerve (LFCN) injury, which is often associated with the DAA [30]. For example, Chen et al. reported that four hips in the DAA group experienced LFCN injury, highlighting a potential drawback of this approach [9]. Additionally, it's worth noting that PA could be more associated with dislocations [31,32]. In our cohort, four [9,14,18,19] of the included studies reported at least one dislocation in the PA group, while no dislocations were observed in the DAA group. These findings emphasize the importance of considering both short-term and long-term complications when evaluating the benefits and drawbacks of each surgical approach.

The considerable heterogeneity (I² = 61%) in blood loss assessment may be attributed to the different methodologies used by each institution. A leave-one-out analysis revealed that when Sarpong et al. were omitted from the analysis, the heterogeneity increased from 61.4% to 78.9% [17]. Sarpong et al. quantified blood loss using the change in hemoglobin levels from preoperative to postoperative day 1, an indirect measure of blood loss [17]. In contrast, Chen et al. directly measured intraoperative blood loss by quantifying suction drain contents and weighing surgical sponges [14]. Unfortunately, Chen et al. did not specify their method of blood loss measurement [9].

Study Limitations

Our study has several limitations. First, the observational design of the included studies, without randomized clinical trials (RCTs) comparing DAA to PA in the context of SimBTHA, limits the strength of our conclusions. Additionally, inconsistent follow-up durations across studies hinder our ability to evaluate long-term outcomes and complications effectively. Variability in complication reporting, with some studies omitting key data, likely leads to an underestimation of adverse events. Moreover, the lack of standardized outcome measures, such as blood loss quantification, introduces bias and limits the ability to compare results across studies directly. This inconsistency makes it difficult to draw robust conclusions and highlights the need for more rigorous research methodologies. These limitations highlight the need for more standardized, well-conducted RCTs to provide stronger evidence and clearer conclusions.

## Conclusions

This systematic review and meta-analysis compared perioperative outcomes between DAA and PA in SimBTHA. The findings revealed no significant differences in allogeneic transfusion rates between the approaches, although DAA was associated with significantly reduced blood loss. Overall complication rates were comparable between the two techniques. Further studies with standardized methodologies and extended follow-up are necessary to validate these results and provide clearer guidance on the optimal surgical approach for SimBTHA.
